# Lipid metabolism-related gene expression pattern of Atlantic bluefin tuna (*Thunnus thynnus* L.) larvae fed on live prey

**DOI:** 10.1007/s10695-016-0305-4

**Published:** 2016-11-04

**Authors:** Mónica B. Betancor, Aurelio Ortega, Fernando de la Gándara, Douglas R. Tocher, Gabriel Mourente

**Affiliations:** 1 Institute of Aquaculture, University of Stirling, Stirling, Scotland FK9 4LA UK; 2 Planta Experimental de Cultivos Marinos, Instituto Español de Oceanografía (IEO), 30860 Puerto de Mazarrón (Murcia), Madrid, Spain; 3 Departamento de Biología, Facultad de Ciencias del Mar y Ambientales, Universidad de Cádiz, 11510 Puerto Real, Cádiz, Spain

**Keywords:** Bluefin tuna, Larvae, Rotifer, Copepods, Lipid content, Lipid classes, Fatty acid composition, cDNA, Gene expression

## Abstract

The present study is the first to evaluate lipid metabolism in first-feeding Atlantic bluefin tuna (ABT; *Thunnus thynnus* L.) larvae fed different live prey including enriched rotifers *Brachionus plicatilis* and *Acartia* sp. copepod nauplii from 2 days after hatch. Understanding the molecular basis of lipid metabolism and regulation in ABT will provide insights to optimize diet formulations for this high-value species new to aquaculture. To this end, we investigated the effect of dietary lipid on whole larvae lipid class and fatty acid compositions and the expression of key genes involved in lipid metabolism in first feeding ABT larvae fed different live prey. Additionally, the expression of lipid metabolism genes in tissues of adult broodstock ABT was evaluated. Growth and survival data indicated that copepods were the best live prey for first feeding ABT and that differences in growth performance and lipid metabolism observed between larvae from different year classes could be a consequence of broodstock nutrition. In addition, expression patterns of lipid metabolic genes observed in ABT larvae in the trials could reflect differences in lipid class and fatty acid compositions of the live prey. The lipid nutritional requirements, including essential fatty acid requirements of larval ABT during the early feeding stages, are unknown, and the present study represents a first step in addressing these highly relevant issues. However, further studies are required to determine nutritional requirements and understand lipid metabolism during development of ABT larvae and to apply the knowledge to the commercial culture of this iconic species.

**Electronic supplementary material:**

The online version of this article (doi:10.1007/s10695-016-0305-4) contains supplementary material, which is available to authorized users.

## Introduction

Improvement in the production of Atlantic bluefin tuna (ABT; *Thunnus thynnus* L) larvae and juveniles is essential to establish full-cycle culture technology for this species. The supply of viable eggs and optimizing the nutritional value of live prey (e.g., rotifers, *Artemia*, copepods, fish yolk-sac larvae) and juvenile diets are paramount to achieve this goal. To date, standard live feeds and artificial diet feeding protocols for larvae and juvenile ABT are giving poor survival and growth and stress resistance. Moreover, size variation, low swimbladder inflation rates, skeletal anomalies, and tank wall collisions are common, not only in ABT culture but also in other bluefin tunas species (Yasunori 2012).

In fish, lipids and their constituent fatty acids (FAs) play essential roles in maintaining optimum growth, survival, feed efficiency, health, neural and visual development, and response to stressors in addition to generally being the main energy source (Sargent et al. 1989, 2002; Tocher, 2003, 2010). Among the lipids and their constituents, phospholipids as well as omega 6 and 3 (n-6 and n-3, respectively) long-chain polyunsaturated fatty acids (LC-PUFA) are particularly important due to their critical roles in the physiological processes above. Indeed, the limited global supply of the n-3 LC-PUFA, eicosapentaenoic acid (20:5n-3; EPA) and docosahexaenoic acid (22:6n-3; DHA) (Tacon and Metian 2008; Tocher 2015), is a major issue for the culture of top predator species such as ABT, which makes it critical to understand the mechanisms by which fish allocate energy from lipids for metabolism, development, growth, and reproduction. Appropriate uptake and accumulation of lipids improve growth and survival of all fish, but, in particular, lipids are relatively more important and key in highly active migratory fish species such as tunas (Mourente and Tocher 2003, 2009) given that the fish obtain energy for the migrations from flesh lipid reserves (Clay 1988).

A key characteristic of tissue FA compositions in large tuna species is that they display high DHA contents (>20 %) and high DHA/EPA ratios (Mourente et al. 2002; Mourente and Tocher 2003, 2009; Ortega and Mourente 2010). Besides, in ABT and other regionally endothermic active-migratory species, the DHA content and DHA/EPA ratio of muscle are much higher than in non-migratory teleost species (Nakamura et al. 2007; Osako et al. 2009). This may also suggest that tuna species may have a dietary requirement for high DHA and a high DHA/EPA ratio. However, there is selective utilization of monoenoic and saturated FAs relative to PUFA as energy sources in tuna, and so the high DHA/EPA ratio could also indicate a selective catabolism of EPA relative to DHA (Mourente and Tocher 2003, 2009; Scholefield et al. 2015). Furthermore, the capacity for endogenous synthesis of EPA and DHA is limited in ABT, and so the lipid biochemistry underpinning the high tissue DHA and DHA/EPA ratio is unclear (Gregory et al. 2010; Morais et al. 2011; Scholefield et al. 2015).

Recently, we investigated lipid and FA metabolism during early development of yolk-sac ABT larvae including the cloning and functional characterization of complementary DNAs (cDNAs) for key enzymes involved in LC-PUFA synthesis, fatty acyl desaturase 6 (*fads2d6*), and elongase of very long-chain fatty acids 5 (*elovl5*) (Morais et al. 2011). In unfed larvae, the level of DHA was maintained until 4 days after hatch (dah), but the proportion of EPA declined, and so the DHA/EPA ratio increased during yolk-sac utilization. As described above, this could be due to the relative retention of DHA during a period of high FA oxidation and utilization, but it was noteworthy that there was also increased expression of *fads2d6* and *elovl5* with larval development potentially leading to endogenous synthesis of DHA (Morais et al. 2011). This suggested that increased activity of these enzymes could be crucial for normal development of ABT larvae, possibly related to the provision of sufficient DHA for the formation of membranes, particularly in neural tissues (Mourente 2003). Hence, studies that emphasize FA metabolism and LC-PUFA synthesis/deposition in different tissues and the transcriptional control mechanisms that regulate these processes are key to understanding lipid nutrition in this species.

The regulation of lipid homeostasis in fish is a complex balance between lipid uptake, transport, storage, energy utilization, and biosynthesis with each process being controlled independently and also in conjunction with other processes (Leaver et al. 2008; Tocher 2003). Recent studies investigating global gene expression using transcriptomic and proteomic approaches have shown that dietary lipid content and composition have significant effects on gene expression in salmonids (Kolditz et al. 2008; Panserat et al. 2008; Higgs et al. 2009; Martinez-Rubio et al. 2013), flatfish (Cho et al. 2009, 2012; Cunha et al. 2013; Peng et al. 2014; Yuan et al. 2015), and other marine species (Tsai et al. 2008; Dong et al. 2015; Li et al. 2015, 2016), as well as Pacific bluefin tuna (PBT) (Agawa et al. 2012). Thus, studying the impact of dietary lipid on lipid and FA metabolism, including effects on whole larvae lipid and FA compositions and the expression of genes of major lipid metabolic pathways including lipogenesis, lipid deposition, FA β-oxidation, and LC-PUFA synthesis in ABT, is highly relevant (Leaver et al. 2008). Furthermore, key to this understanding is knowledge of the lipid-regulated transcription factors (TFs) and nuclear receptors controlling and regulating the expression of genes involved in FA/lipid metabolic pathways. In this sense, studies in mammals established that members of the peroxisome proliferator-activated receptor (PPAR), liver X receptor (LXR), and sterol regulatory element binding protein (SREBP) TFs control an integrated network of lipid and FA metabolism (Nakamura et al. 2004).

The aims of the present study were to investigate the effect of dietary lipid on lipid and fatty acid compositions as well as on the expression of key genes involved in lipid metabolism in ABT larvae fed different live prey. Specific objectives were first to clone cDNAs of ABT genes involved in major lipid metabolism pathways and their control and regulation, including fatty acid and LC-PUFA biosynthesis, lipid deposition, and β-oxidation, for the evaluation of gene expression. The second aim is to determine the expression of these genes in first feeding ABT larvae 14 dah, and third, to determine the expression of these genes and the major lipid pathways in tissues of adult ABT. Our overarching hypothesis is that understanding the molecular basis of lipid metabolism and regulation will provide insight to optimize diet formulations and the effective use of sustainable dietary lipid sources in ABT aquaculture.

## Materials and methods

### Atlantic bluefin tuna larvae rearing conditions

The ABT larvae used in this study were obtained from two consecutive larval rearing trials performed in July 2013 and July 2014, respectively. The ABT eggs were obtained from a broodstock composed of 35 fish with an estimated mean body weight of 100 kg. The broodstock were maintained in captivity for several years in a floating cage located at El Gorguel Bay, off Cartagena coast, South East Spain. Captive ABT broodstock fish spawned naturally and spontaneously (during the natural spawning season in June–July). A 1.5-m polyvinyl sheet was placed around the inside of the cage to avoid eggs drifting away from the cage by means of currents or waves and floating eggs collected inside the cage by means of a net of 500-μm mesh screen size. Collected eggs were transported in a 500-L plastic tank supplied with pure oxygen to the IEO Planta Experimental de Cultivos Marinos (Puerto de Mazarrón, Murcia, Spain) aquaculture facilities and placed in 100-L tanks with gentle aeration and flow-through sterilized seawater. After 1 h, aeration and water flow were stopped to separate buoyant (viable) from non-buoyant (non-viable) eggs. After washing and counting, the eggs were incubated in 1500-L cylindrical tanks at a density of 10 eggs L^−1^. Incubation was carried out at 25–26 °C, 37 ‰ salinity, and continuous photoperiod, with a light intensity of 1000 lx. An upwelling flow-through with gentle aeration was employed in order to maintain oxygen levels near to saturation. Larvae hatched approximately 32 h after fertilization, with a hatching rate of almost 90 %, and were fed with rotifers or copepod nauplii 2 dah. A mixture of the microalgae *Isochrysis* sp. (T-Iso, Thaitian strain of *Isochrysis*) and *Chlorella* (V12 DHA-enriched, Pacific Trading Co., Japan) was added to tanks at a density of 200–300,000 cells mL^−1^ as green water. During the trials, photoperiod was maintained at 14/10 h light/dark (light intensity about 1000 lx), temperature ranged between 22.9 and 24.8 °C, and daily water renewal was 50–70 %. Incoming seawater was filtered through a 10-μm sieve and UV sterilized. An upwelling current was created to avoid larvae sinking (mainly at night) and maintain oxygen level.

1. *Larval trial 2013*. Two different feeding treatments were tested from the beginning of exogenous feeding 2 to 14 dah: (i) L-type rotifers (*Brachionus plicatilis*), cultured with DHA-enriched *Chlorella* (*Chlorella* V12, Pacific Trading Co.) and enriched with taurine (500 ppm, added to rotifer culture tanks during 18 h before harvesting) and Skretting® ORI-Green over 3 h at a dose of 0.3 g per million rotifers, and (ii) nauplii of the copepod *Acartia grani* fed on *Isochrysis* T-Iso and copepodites fed on a mixture of *Isochrysis* T-Iso and *Tetraselmis* sp. To maintain constant live prey concentration (10 rotifer mL^−1^ or five copepod nauplii/copepodite mL^−1^) within each experimental tank, three water samples (10 mL) from each tank were sampled and counted before supplying new feed.
2. *Larval trial 2014.* Three feeding regimes were tested 2 to 14 dah: (i) enriched L-type rotifers (*B. plicatilis*) supplemented with taurine and Skretting® ORI-Green as above, (ii) nauplii of the copepod *Acartia tonsa* as above, and (iii) co-feeding (50:50) of enriched rotifers and *Acartia* nauplii. *Acartia tonsa* eggs were incubated at 30 ‰ salinity and 25–26 °C and, after hatching, were fed on *Rhodomonas baltica* and *Isochrysis aff. galbana* T-Iso for 2–4 days. Density of the live prey was as follows: rotifers were maintained at 10 rotifers per milliliter, *A. tonsa* at five nauplii ^—^per milliliter, and co-feeding of rotifers and *Acartia* at five individuals per milliliter as a 50 % mixture of both organisms.

### Sample collection

Larvae (20 for each rearing condition) were anesthetized (0.02 % phenoxyethanol) and total lengths measured. Replicates of preweighed samples (approximately 50 mg wet weight) were maintained at 110 °C for 24 h and dry weights determined after cooling in vacuo for 1 h. Triplicate samples of rotifers and copepods (*Acartia*) were washed and filtered, excess water drained and blotted with filter paper, immediately frozen in liquid N_2_ and stored at −80 °C prior to lipid analysis. Two subsets of triplicate samples of 14 dah ABT larvae fed the different live prey used in 2013 and 2014 feeding trials were collected. One subset of samples was placed in 1 mL of RNA*later*® (Ambion, Madrid, Spain) for RNA extraction, and a second subset was frozen in liquid N_2_ and stored at −80 °C for lipid analysis. Eight broodstock tuna (four males and four females), culled for reproductive stage assessment, were used for collecting tissue samples for tissue expression of lipid metabolism genes. After sacrifice, triplicate sets of samples of brain, gills, heart, kidney, spleen, liver, intestine, white muscle, red muscle, adipose tissue ovary, and testis were collected. Each replicate (about 100 mg) was placed in 1 mL of RNA*later*® (Ambion), stored at 4 °C overnight before transferring to −20 °C, and subsequently stored prior to RNA extraction. All procedures were carried out according to the current Spanish and European Union legislation on the handling of experimental animals.

### Lipid content, lipid class composition, and fatty acid analysis

Total lipid of live feeds (enriched rotifers and copepods) and ABT larvae fed the different regimes was extracted from triplicate pooled samples according to the method of Folch et al. (1957). Approximately 1 g of ABT larvae was placed in 20 mL of ice-cold chloroform/methanol (2:1, by vol.) and homogenized with an Ultra-Turrax tissue disrupter (Fisher Scientific, Loughborough, UK). The non-lipid and lipid layers were separated by addition of 5 mL of 0.88 % (*w*/*v*) KCl and allowed to separate on ice for 1 h. The upper non-lipid layer was aspirated and the lower lipid layer evaporated to dryness under oxygen-free nitrogen. The lipid content was determined gravimetrically after drying overnight in a vacuum desiccator.

Lipid class composition was determined by high-performance thin-layer chromatography (HPTLC) using 10 × 10-cm plates (VWR, Lutterworth, England). Approximately 1 μg of total lipid was applied as a single spot and the plates developed in methyl acetate/isopropanol/chloroform/methanol/0.25 % aqueous KCl (25:25:25:10:9, by vol.) to two thirds up the plate. After drying for 20 min, the plate was fully developed with isohexane/diethyl ether/acetic acid (85:15:1, by vol.). The lipid classes were visualized by charring at 160 °C for 15 min after spraying with 3 % (*w*/*v*) aqueous cupric acetate containing 8 % (*v*/*v*) phosphoric acid and quantified by densitometry using a CAMAG-3 TLC Scanner (version Firmware 1.14.16) (Henderson and Tocher 1992). Scanned images were recorded automatically and analyzed by computer using winCATS Planar Chromatography Manager (version 1.2.0).

Fatty acid methyl esters (FAMEs) were prepared from total lipid by acid-catalyzed transesterification at 50 °C for 16 h according to the method of Christie (1993). The FAME were separated and quantified by gas-liquid chromatography (Carlo Erba Vega 8160, Milan, Italy) using a 30 m × 0.32-mm i.d. capillary column (CP-Wax 52CB, Chrompak, London, UK) and on-column injection at 50 °C. Hydrogen was used as carrier gas, and temperature programming was from 50 to 150 °C at 40 °C min^−1^ and then to 230 °C at 2.0 °C min^−1^. Individual methyl esters were identified by comparison with known standards and by reference to published data (Ackman 1980; Tocher and Harvie 1988). Data were collected and processed using Chromcard for Windows (version 1.19).

### Tissue RNA extraction and cDNA synthesis

Approximately 100 mg of pooled larvae (*n* = 3 per treatment) and adult ABT tissues from eight individuals (see above) were placed in RNA*later*® (Sigma-Aldrich, Dorset, UK) and frozen at −20 °C for total RNA extraction. Samples were homogenized in 1 mL of TRI Reagent® (Sigma-Aldrich) RNA extraction buffer using a bead tissue disruptor (BioSpec, Bartlesville, OK, USA). Total RNA was isolated following manufacturer’s instructions and quantity and quality determined by spectrophotometry using a NanoDrop ND-1000 (Labtech Int., East Sussex, UK) and electrophoresis using 200 ng of total RNA in 1 % agarose gel. cDNA was synthesized using 2 μg of total RNA and random primers in 20-μL reactions and the High Capacity Reverse Transcription Kit without RNase inhibitor according to the manufacturer’s protocol (Applied Biosystems, Warrington, UK).

### Sequencing of genes of interest

Several genes related to lipid and fatty acid metabolism were evaluated in the present study. Quantitative real-time PCR (qPCR) was carried out on cDNAs encoding the transcription factors *pparα*, *pparγ*, *lxr*, *rxr*, *srebp1*, and *srebp2*; fatty acid biosynthesis genes *fads2d6* and *elovl5*; and fatty acid metabolism genes *fas*, *cpt1*, *aco*, *fabp2*, *fabp4*, *fabp7*, *lpl*, and *hmgcl* (Supplementary Table 1). Sequences corresponding to the open reading frame (ORF) of *srebp1*, *srebp2*, *pparα*, *lxr*, and *cptI* from several fish species were aligned, and primers were designed on common conserved regions. GenBank accession numbers of the sequences used in these alignments were JF502069.1 (*Siganus canaliculatus*), XM_010874222.1 (*Esox lucius*), XM_008287928.1 (*Stegastes partitus*), and XM_005734603.1 (*Pundamilia nyererei*) for *srebp1*; NM_01195819.1 (*Salmo salar*), XM_008300413.1 (*S. partitus*), XM_005468438.1 (*Oreochromis niloticus*), XM_005940738.1 (*Haplochromis burtoni*), XM_004563041.1 (*Maylandia zebra*), and XM_006797937.1 (*Neolamprologus brichardi*) for *srebp2*; NM_001161333.1 (*Danio rerio*), FJ231987.1 (*Ctenopharyngodon idella*), FJ849065.1 (*Cyprinus carpio*), NM_001123560.1 (*S. salar*), NM_001197211.1 (*Oncorhynchus mykiss*), AB298547.1 (*Pagrus major*), JN971011.1 (*Oncorhynchus nerka*), FJ208989.1 (*Lateolabrax japonicus*), JX975469.1 (*Scophthalmus maximus*), JQ707899.1 (*Synechogobius ommaturus*), EU195886.1 (*Pimephales promelas*), and HM140628.2 (*Megalobrama amblycephala*) for *pparα*; NM_001145421.1 (*S. salar*), NM_001159338.1 (*O. mykiss*), AB759916.1 (*Paralichthys olivaceus*), FJ965309.2 (*C. idella*), FJ919778.1 (*C. carpio*), and NM_001017545.1 (*D. rerio*) for *lxr*; and HM037343.1 (*Epinephelus coioides*), JQ707894.1 (*S. ommaturus*), NM_001246330.1 (*O. mykiss*), and JQ663626.1 (*Sebasticus marmoratus*) for *cptI*. Fragments were obtained by reverse-transcription PCR (MyTaq™ HS Mix, Bioline, London, UK) from cDNA (High Capacity Reverse Transcription Kit; Applied Biosystems, Warrington, UK) obtained from 2 μg of total RNA pooled from adult ABT tissues and the primers designed for the conserved regions for each gene. PCR products were ligated into plasmid pCR2.1 (TA Cloning Kit, Invitrogen, Paisley, UK) and sequenced (Sanger ABI3730xl, GATC Biotech, Konstanz, Germany) and primers for qPCR designed.

Sequences for *fads2d6* and *elovl5* were already available for ABT (Morais et al. 2011). Primers for *fabp2*, *4*, and *7*; *rxr*; and *hmgl* were designed on existing expressed sequence tags (ESTs) derived from ABT liver, ovaries, and testis (EG999641, EC092703, EG999669, EC092909, and EH668469, respectively; Chini et al. 2008). *Pparγ* primers were designed on the *pparγ* sequence of *Thunnus orientalis* (AB574331.1). Primers for *aco*, *fas*, and *lpl* were designed on sequences included in an ABT oligonucleotide DNA microarray (ArrayExpress database accession number E-MTAB-3412; Trumbić et al. 2015).

### qPCR analysis

Expression of genes of interest was determined by qPCR of all the RNA samples. Elongation factor-1α (*elf1α*) and *β-actin* were used as reference genes to study nutritional regulation. The cDNA was diluted 20-fold with Milli-Q water. The efficiency of the primers for each gene was previously evaluated by serial dilutions of cDNA pooled from the samples to guarantee it was >85 % for all primer pairs. qPCR was performed using a Biometra TOptical Thermocycler (Analytik Jena, Goettingen, Germany) in 96-well plates in duplicate 20-μL reaction volumes containing 10 μL of Luminaris Color HiGreen qPCR Master Mix (Thermo Scientific, Hemel Hempstead, UK), 1 μL of the primer corresponding to the analyzed gene (10 pmol), 3 μL of molecular biology grade water, and 5 μL of cDNA (1/20 diluted). In addition, amplifications were carried out with a systematic negative control (NTC, no template control) containing no cDNA. Standard amplification parameters contained a UDG pretreatment at 50 °C for 2 min and an initial denaturation step at 95 °C for 10 min, followed by 35 cycles: 15 s at 95 °C, 30 s at the annealing temparature, and 30 s at 72 °C. The relative expression of each gene among the tissues was calculated as arbirary units after normalization against the expression level of the housekeeping gene *elf1α*. One arbitrary unit was equal to the expression level of the gene expressed at the lowest level per each set of genes.

### Statistical analysis

Results for biometry, lipid class, and fatty acid compositions are presented as means ± SD (*n* = 20 for biometry and *n* = 3 for survival, lipid class, and fatty acid compositions). The data were checked for homogeneity of the variances by the Bartlett test and, where necessary, arcsine-transformed before further statistical analysis. Differences between mean values were analyzed by *t* test and one-way analysis of variance (ANOVA), followed when pertinent by a multiple comparison test (Tukey). Differences were reported as statistically significant when *P* < 0.05 (Zar 1999). Gene expression results were analyzed using the relative expression software tool (REST 2009), which employs a pairwise fixed reallocation randomization test (10,000 randomizations) with efficiency correction (Pfaffl et al. 2002) to determine the statistical significance of expression ratios (gene expression fold changes) between two treatments. In addition, a supervised hierarchical clustering was applied employing the relative gene expression ratio for each gene based on the PCR efficiency and Cycle threshold (Ct) of sample compared to the control, according to Pfaffl’s mathematical model (Pfaffl 2001).

## Results

### ABT larvae biometry and survival in feeding trials

Total length, individual dry mass, and survival of 14-dah ABT larvae are shown in Table 1. In the 2013 trial, total length of ABT larvae fed on enriched rotifers was significantly greater than that of larvae fed on copepods, although total dry mass was not significantly different. However, survival was nearly 2-fold higher in the ABT larvae fed copepods. In 2014, total length and total dry mass were highest for ABT larvae fed copepods and lowest in larvae fed rotifers, with intermediate values in larvae co-fed with rotifers and copepods. However, survival was highest in co-fed larvae followed by larvae fed copepods and rotifers, respectively.

**Table 1:** Rearing performance of 14-day after hatch Atlantic Bluefin tuna (*Thunnus thynnus*) larvae fed enriched rotifers *Brachionus plicatilis*, *Acartia* sp. copepod nauplii, and co-feeding rotifer + copepod in 2013 and 2014 feeding trials

	Trial	2013		2014		
		ABT + rotifer	ABT + copepod	ABT + rotifer	ABT + copepod	ABT + rotifer + copepod
	Total length (mm)	7.75 ± 0.61	7.26 ± 0.51*	7.02 ± 0.17^c^	8.29 ± 0.08^a^	7.50 ± 0.22^b^
	Dry mass (mg)	0.66 ± 0.13	0.61 ± 0.13	0.35 ± 0.03^c^	0.77 ± 0.01^a^	0.51 ± 0.06^b^
	Survival (%)	3.18 ± 1.12	5.91 ± 0.93*	2.88 ± 1.02^c^	7.49 ± 1.17^b^	10.24 ± 3.50^a^

Results are means ± SD (*n* = 20 for total length and dry mass and *n* = 3 for survival). Different superscript letters denote significant difference (*P* < 0.05) for one-way ANOVA and Tukey multiple comparison tests in 2014 trial

*ABT* Atlantic bluefin tuna

*Significant difference (*p* < 0.05) for t tests analysis in 2013 trial

### Total lipid content and lipid class composition of the live feeds and the ABT larvae

Total lipid content of the enriched rotifers was 13–16 % of dry mass and significantly higher than the total lipid content of the copepods (6–9.5 % of dry mass) in the two trials (Tables 2 and 3). The lipid content of the ABT larvae fed rotifers was 1.0–1.2 % for both years whereas the larvae fed the copepods or co-fed the rotifers and the copepods had a total lipid content that was 0.8 % of live mass, although these differences were not significant (Tables 2 and 3).

**Table 2:** Trial in 2013—total lipid (% dry mass for prey and % live mass for ABT larvae) and lipid class composition (% of total lipid) of (a) rotifer *Brachionus plicatilis* enriched with Skretting® ORI-Green, (b) nauplii of the copedod *Acartia granii* fed *Isocrysis* T-Iso, and 14-day after hatch Atlantic bluefin tuna (ABT) larvae fed rotifers (c) and copepods (d)

	(a) Rotifer	(b) Copepod	(c) ABT + rotifer	(d) ABT + copepod
Total lipid (% dry mass)	13.4 ± 0.9	6.5 ± 0.3*	1.0 ± 0.1	0.8 ± 0.2
Lipid class				
Phosphatidylcholine	14.7 ± 0.5	14.2 ± 1.5	25.0 ± 0.8	22.2 ± 1.4
Phosphatidylethanolamine	13.3 ± 0.5	6.8 ± 1.4*	11.4 ± 0.6	11.0 ± 0.3
Phosphatidylserine	4.9 ± 0.2	1.6 ± 0.9*	7.3 ± 0.9	9.5 ± 0.7*
Phosphatidylinositol	7.7 ± 0.5	1.3 ± 0.1*	3.7 ± 1.1	4.6 ± 0.6
Phosphatidic acid/cardiolipin	nd	3.5 ± 0.8	3.1 ± 0.1	2.0 ± 0.2*
Sphingomyelin	0.5 ± 0.0	5.2 ± 0.1*	2.3 ± 0.4	2.8 ± 0.3
Lyso-phosphatidylcholine	2.4 ± 0.1	3.5 ± 0.2*	1.8 ± 0.3	1.6 ± 0.1
Total polar lipids	55.9 ± 1.5	50.5 ± 4.9	66.4 ± 2.0	63.2 ± 1.7
Cholesterol	4.1 ± 0.6	14.2 ± 0.7*	10.6 ± 0.3	14.9 ± 0.9*
Triacylglycerol	25.5 ± 0.9	25.5 ± 3.9	13.8 ± 0.9	11.6 ± 1.8
Steryl/wax ester	5.4 ± 0.2	1.0 ± 0.1*	3.1 ± 0.9	5.0 ± 0.5
Free fatty acid	8.9 ± 0.3	8.4 ± 0.4	5.9 ± 0.1	5.0 ± 1.3
Total neutral lipids	44.1 ± 1.5	49.5 ± 4.9	33.6 ± 2.0	36.8 ± 1.7
Triacylglycerol:cholesterol	6.2 ± 0.7	1.8 ± 0.2 *	1.3 ± 0.1	0.8 ± 0.2

Results are means ± SD (*n* = 3)

*Significant difference (*P* < 0.05) for *t* test analysis

**Table 3:** Trial in 2014—total lipid (% dry mass for prey and % live mass for ABT larvae) and lipid class composition (% of total lipid) of rotifer *Brachionus plicatilis* enriched with Skretting® ORI-Green (a), nauplii of the copedod *Acartia tonsa* fed on *Rhodomonas salina* and *Isochrysis* T-Iso (b), and 14-day after hatch Atlantic bluefin tuna (*Thunnus thynnus*) larvae fed rotifers (c), copepod (d), and rotifer + copepod (e)

	(a) Rotifer	(b) Copepod	(c) ABT + rotifer	(d) ABT + copepod	(e) ABT + rotifer + copepod
TL (% dry mass)	15.9 ± 1.6	9.0 ± 0.6*	1.2 ± 0.0	0.8 ± 0.1	0.8 ± 0.3
Lipid class					
Phosphatidylcholine	12.0 ± 0.5	16.2 ± 0.5*	22.2 ± 0.7	20.4 ± 0.2	21.6 ± 1.0
Phosphatidylethanolamine	13.2 ± 0.5	7.7 ± 0.3*	14.2 ± 1.5	13.9 ± 0.5	13.5 ± 1.0
Phosphatidylserine	4.9 ± 0.8	6.2 ± 0.5	8.6 ± 0.4^a^	7.5 ± 0.6^ab^	6.7 ± 0.6^b^
Phosphatidylinositol	8.8 ± 0.6	5.1 ± 0.3*	5.7 ± 0.3^a^	4.4 ± 0.2^b^	4.0 ± 0.4^b^
Phosphatidic acid/cardiolipin	1.6 ± 0.2	3.9 ± 0.1*	1.5 ± 0.1	1.6 ± 0.2	1.7 ± 0.2
Sphingomyelin	0.5 ± 0.1	5.6 ± 0.4*	2.6 ± 0.2	2.1 ± 0.2	2.0 ± 0.2
Lyso-phosphatidylcholine	1.8 ± 0.3	2.4 ± 0.2	0.6 ± 0.1^a^	0.6 ± 0.1^a^	0.9 ± 0.1^b^
Total polar Lipids	52.8 ± 1.7	55.8 ± 0.3	67.3 ± 1.1^a^	62.6 ± 0.2^b^	62.8 ± 1.9^ab^
Cholesterol	11.2 ± 1.2	10.9 ± 0.3	10.0 ± 0.6	11.0 ± 0.4	11.0 ± 0.3
Triacylglycerol	23.6 ± 2.1	20.6 ± 0.5	13.6 ± 0.7^a^	15.0 ± 0.4^ab^	17.6 ± 2.8^b^
Steryl/wax ester	5.6 ± 0.3	1.7 ± 0.1*	3.6 ± 0.2^a^	6.1 ± 0.6^b^	3.2 ± 0.8^a^
Free fatty acid	6.7 ± 1.1	10.9 ± 0.4*	5.5 ± 0.2	5.2 ± 0.2	5.1 ± 0.8
Total neutral Lipids	47.4 ± 1.7	44.2 ± 0.3	32.7 ± 1.1^a^	37.4 ± 0.2^b^	37.2 ± 1.9^b^
TAG/C	2.1 ± 0.3	1.9 ± 0.1	1.4 ± 0.1	1.4 ± 0.1	1.6 ± 0.3

Results are mean ± SD (*n* = 3). A different superscript letter denotes significantly different (*p* < 0.05) for one-way ANOVA and Tukey multiple comparison tests in 2014 trial

*TAG/C* triacylglycerol/colesterol ratio. *ABT* Atlantic bluefin tuna

*indicates significantly different (*p* < 0.05) for t-tests analysis among the live preys

In 2013, the lipids of the enriched rotifers were predominantly polar lipids (∼56 %), mainly phosphatidylcholine (PC, ∼ 15 %) and phosphatidylethanolamine (PE, ∼ 13 %), with total neutral lipids accounting for 44 %, primarily triacylglycerol (TAG, ∼ 25 %) (Table 2). Compared to the rotifers, the copepods in 2013 had lower proportions of PE, phosphatidylserine (PS), phosphatidylinositol (PI), and steryl ester (SE) and higher proportions of sphingomyelin (SM), phosphatidic acid/cardiolipin, and cholesterol. Irrespective of the live feed used, the lipid class composition of the ABT larvae showed relative high levels of polar lipids (63–66 %) with high proportions of PC (22–25 %) followed by PE (11 %), and the feed had relatively little effect on the larvae lipid class, although larvae fed copepods showed higher proportions of PS and cholesterol than larvae fed rotifers (Table 2). In 2014, the lipid class composition of the enriched rotifers was very similar to that in 2013, but that of the copepods was slightly different (Table 3). However, the differences between the rotifers and the copepods were largely similar to those in 2013 with copepods showing lower proportions of PE and PI and higher proportions of PC, SM, phosphatidic acid/cardiolipin, and free fatty acid compared to the rotifers (Table 3). As in the 2013 trial, the live feeds had little impact on the lipid class composition of the ABT larvae. Larvae fed rotifers had higher proportions of polar lipids (∼67 %) compared to larvae fed copepods or co-fed rotifers and copepods (∼ 62 %), due to increased percentages of all phospholipid classes, especially PE and PS, and lower proportions of TAG and SE (Table 3). Co-fed larvae showed a very similar lipid class profile to those fed copepods.

### Total lipid fatty acid compositions of the live feeds and the ABT larvae

Total lipids of rotifers in 2013 were characterized by 25 % saturated fatty acids (primarily 16:0 followed by 18:0), almost 13 % monounsaturated fatty acids (MUFA), primarily 18:1n-9, and around 60 % PUFA, primarily 18:2n-6 (∼21 %) with 18:3n-3 (∼7 %) (Table 4). Copepods showed similar levels of saturated fatty acids albeit with higher proportions of 14:0 and lower proportions of 16:0. Additionally, similar proportions of MUFA (higher 16:1n-7 and lower 20:1n-9) and lower 18:2n-6 and 18:3n-3 but higher 18:4n-3 and n-3:n-6 PUFA ratio were observed in copepods compared to rotifers (Table 4). Besides, rotifers and copepods provided similar DHA/EPA ratios (2.7 and 2.8, respectively) but percentages of DHA and EPA were twice as high in copepods (25 and 9 %, respectively) compared to rotifers (12 and 4.5 %, respectively). Higher proportions of 22:5n-3 were found in rotifers compared to copepods.

**Table 4:** Total lipid fatty acid composition (weight %) of rotifers *B. plicatilis* enriched with Skretting® ORI-Green (a), nauplii of the copepod *Acartia granii* fed on *Rhodomonas salina* (b), and 14-day after hatch Atlantic bluefin tuna (*Thunnus thynnus* L.) larvae fed with rotifers (c) and copepods (d) in the 2013 trial

	Live preys		ABT larvae	
	(a)	(b)	(c)	(d)
Fatty acid				
14:0	0.9 ± 0.1	8.9 ± 0.4*	0.8 ± 0.0	1.8 ± 0.3*
16:0	19.6 ± 0.2	14.9 ± 0.7*	18.4 ± 0.4	19.2 ± 0.8
18:0	3.4 ± 0.1	2.9 ± 0.1*	9.3 ± 0.4	9.5 ± 1.4
Total saturated^a^	25.0 ± 0.2	27.2 ± 0.6*	30.1 ± 0.3	31.9 ± 1.3
16:1n-7	0.8 ± 0.0	5.1 ± 0.1*	1.2 ± 0.3	2.5 ± 0.1*
18:1n-9	4.5 ± 0.1	5.2 ± 0.1*	6.3 ± 0.9	6.0 ± 0.2
18:1n-7	0.9 ± 0.1	1.3 ± 0.2	1.6 ± 0.2	2.2 ± 0.1
20:1n-9	2.1 ± 0.3	0.3 ± 0.1*	1.3 ± 0.2	0.4 ± 0.1*
Total monoenes^b^	12.7 ± 0.2	13.3 ± 0.5	14.6 ± 0.9	12.7 ± 0.3
C16 PUFA	6.5 ± 0.3	0.7 ± 0.1*	2.6 ± 0.1	1.9 ± 0.2*
18:2n-6	20.5 ± 0.3	8.7 ± 0.2*	13.1 ± 0.5	4.5 ± 0.3*
20:4n-6	0.6 ± 0.0	1.2 ± 0.0*	1.3 ± 0.1	1.5 ± 0.1
22:5n-6	0.5 ± 0.0	2.5 ± 0.1*	0.6 ± 0.1	2.2 ± 0.2*
Total n-6PUFA^c^	27.9 ± 0.3	14.7 ± 0.1*	18.1 ± 0.7	10.5 ± 0.3*
18:3n-3	6.7 ± 0.1	3.9 ± 0.0*	3.0 ± 0.1	1.7 ± 0.2*
18:4n-3	0.2 ± 0.0	3.4 ± 0.1*	0.4 ± 0.0	0.9 ± 0.2*
20:4n-3	1.0 ± 0.1	0.1 ± 0.0*	0.9 ± 0.1	0.6 ± 0.0*
20:5n-3	4.5 ± 0.1	8.9 ± 0.1*	5.4 ± 0.1	5.9 ± 0.4
22:5n-3	3.9 ± 0.2	0.3 ± 0.0*	4.3 ± 0.4	1.0 ± 0.0*
22:6n-3	12.1 ± 0.5	24.9 ± 0.9*	17.5 ± 0.9	29.6 ± 1.0*
Total n-3PUFA^d^	32.1 ± 0.7	42.3 ± 0.9*	33.8 ± 0.4	41.3 ± 1.7*
Total PUFA	60.0 ± 0.7	57.0 ± 3.9*	51.9 ± 1.3	51.8 ± 2.0
n-3/n-6	1.1 ± 0.1	2.9 ± 0.1*	1.9 ± 0.1	3.9 ± 0.1*
DHA/EPA	2.7 ± 0.1	2.8 ± 0.1	3.2 ± 0.2	5.0 ± 0.1*

Results are means ± SD (*n* = 3). An SD of 0.0 implies an SD of <0.05

*DHA* docosahexaenoic acid, *EPA* eicosapentaenoic acid, *PUFA* polyunsaturated fatty acid

*Significantly different (*P* < 0.05)

^a^Totals include 15:0, 20:0, 22:0, and 24:0

^b^Totals include 16:1n-9, 18:1n-11, 20:1n-7, 22:1 isomers, and 24:1

^c^Totals include 18:3n-6, 20:2n-6, 22:4n-6, and 22:5n-6

^d^Totals include 20:3n-3 and 22:3n-3

Total lipid of ABT larvae was about 30–32 % saturated fatty acids and 13–15 % MUFA irrespective of live feed used with few important differences (Table 3). However, the PUFA compositions of the live feeds were reflected in ABT larvae compositions and thus larvae fed copepods showed lower 18:2n-6, 18:3n-3, and 22:5n-3 and higher DHA and n-3:n-6 PUFA than larvae fed rotifers. Interestingly, the proportions of EPA were lower and DHA and the DHA/EPA ratio higher in larvae fed copepods than in the copepods themselves (Table 4).

Rotifers and copepods in 2014 showed similar proportions of saturated fatty acids (27–28 %) and MUFA (∼11 %) as in 2013 with the same differences between the live feeds in the proportions of 14:0, 16:0, 16:1n-7, and 20:1n-9 (Table 5). Similarly, copepods showed lower 18:2n-6 but higher 18:3n-3, 18:4n-3, and n-3/n-6 PUFA ratio than rotifers. In 2014, both rotifers and copepods provided higher DHA/EPA ratios than in 2013 with the ratio being significantly higher in copepods (6.9) compared to rotifers (3.8). As in 2013, the percentage of DHA was higher in copepods but both EPA and 22:5n-3 were higher in rotifers (Table 5).

**Table 5:** Fatty acid composition (weight %) of total lipid of rotifers *B. plicatilis* enriched with Skretting® ORI-Green (a), nauplii of the copepod *Acartia tonsa* fed *Rhodomonas salina* and *Isochrysis* T-Iso (b), and 14-day after hatch Atlantic bluefin tuna (*Thunnus thynnus* L.) larvae fed with rotifers (c), copepods (d), and co-feed rotifer + copepod (e) in the 2014 trial

	Live prey		ABT larvae		
	(a)	(b)	(c)	(d)	(e)
Fatty acid					
14:0	0.7 ± 0.1	11.0 ± 0.2*	0.7 ± 0.1^b^	2.2 ± 0.2^a^	1.6 ± 0.3^a^
16:0	22.7 ± 0.9	11.9 ± 0.2*	16.9 ± 0.3	18.3 ± 0.5	17.1 ± 0.4
18:0	2.9 ± 0.1	2.1 ± 0.0*	8.6 ± 0.4	7.8 ± 0.1	8.3 ± 0.4
Total saturated^a^	28.1 ± 1.2	26.6 ± 0.6	27.9 ± 0.5	31.6 ± 0.7	29.8 ± 0.3
16:1n-7	0.7 ± 0.0	3.9 ± 0.1*	1.7 ± 0.3^ab^	2.3 ± 0.1^a^	1.4 ± 0.1^b^
18:1n-9	4.5 ± 0.3	4.5 ± 0.2	4.9 ± 0.2^b^	5.7 ± 0.2^a^	5.1 ± 0.2^ab^
18:1n-7	0.9 ± 0.1	1.2 ± 0.1	1.9 ± 0.2	2.0 ± 0.1	1.7 ± 0.1
20:1n-9	1.9 ± 0.1	0.3 ± 0.1*	1.2 ± 0.1^a^	0.3 ± 0.0^b^	0.9 ± 0.1^a^
Total monoenes^b^	11.6 ± 0.2	11.8 ± 0.4	12.9 ± 0.3^b^	15.7 ± 0.3^a^	14.0 ± 0.3^ab^
C16 PUFA	3.6 ± 0.3	1.1 ± 0.1*	3.0 ± 0.1^a^	2.2 ± 0.1^b^	2.8 ± 0.1^a^
18:2n-6	14.8 ± 1.1	8.0 ± 0.2*	12.7 ± 0.7^a^	4.2 ± 0.1^b^	10.3 ± 0.9^a^
20:4n-6	0.7 ± 0.0	0.6 ± 0.0	2.6 ± 0.3^a^	1.6 ± 0.1^b^	1.4 ± 0.1^b^
22:5n-6	0.9 ± 0.3	2.6 ± 0.1*	0.4 ± 0.1^c^	2.6 ± 0.2^a^	1.8 ± 0.3^b^
Total n-6PUFA^c^	20.4 ± 1.3	14.1 ± 0.1*	19.4 ± 0.6^a^	14.6 ± 0.2^b^	20.0 ± 1.1^a^
18:3n-3	3.9 ± 0.2	5.2 ± 0.3*	2.3 ± 0.1	2.5 ± 0.2	2.4 ± 0.1
18:4n-3	0.3 ± 0.1	8.2 ± 0.6*	0.2 ± 0.0^c^	2.2 ± 0.2^a^	1.0 ± 0.2^b^
20:4n-3	0.7 ± 0.1	0.1 ± 0.0*	0.7 ± 0.1	0.9 ± 0.2	0.8 ± 0.2
20:5n-3	4.5 ± 0.1	3.8 ± 0.1*	6.6 ± 0.4^a^	5.5 ± 0.1^b^	4.8 ± 0.1^c^
22:5n-3	4.9 ± 0.3	0.3 ± 0.0*	4.6 ± 0.2^a^	0.6 ± 0.0^c^	2.2 ± 0.3^b^
22:6n-3	17.5 ± 3.1	26.0 ± 0.9*	17.6 ± 0.5^b^	28.2 ± 0.3^a^	24.4 ± 0.5^a^
Total n-3PUFA^d^	34.6 ± 3.1	44.9 ± 0.3*	34.5 ± 0.3^b^	44.1 ± 0.3^a^	40.1 ± 0.3^a^
Total PUFA	55.1 ± 1.8	59.0 ± 0.9	53.8 ± 0.6	51.1 ± 2.0	53.7 ± 1.3
n-3/n-6	1.7 ± 0.3	3.2 ± 0.1*	1.7 ± 0.3^b^	3.0 ± 0.1^a^	2.0 ± 0.2^b^
DHA/EPA	3.8 ± 0.6	6.9 ± 0.6*	2.7 ± 0.2^b^	5.1 ± 0.2^a^	5.0 ± 0.2^a^

Results are means ± SD (*n* = 3). An SD of 0.0 implies an SD of <0.05. Values bearing different superscript letters (ABT larvae) are significantly different (*P* < 0.05)

*DHA* docosahexaenoic acid, *EPA* eicosapentaenoic acid, *PUFA* polyunsaturated fatty acid

*Values within live prey are significantly different (*P* < 0.05)

^a^Totals include 15:0, 20:0, 22:0, and 24:0

^b^Totals include 16:1n-9, 18:1n-11, 20:1n-7, 22:1 isomers, and 24:1

^c^Totals include 18:3n-6, 20:2n-6, and 22:4n-6

^d^Totals include 20:3n-3 and 22:3n-3

As in 2013, the PUFA composition of ABT larvae in 2014 tended to reflect the dietary compositions and therefore larvae fed copepods showed lower 18:2n-6, EPA, and 22:5n-3 and higher DHA, DHA/EPA, and n-3/n-6 PUFA ratios than larvae fed rotifers (Table 5). Larvae co-fed rotifers and copepods tended to give intermediate values as observed, for instance, with 18:2n-6, 22:5n-3, DHA, and n-3/n-6 ratio (Table 5).

### Expression of lipid metabolism genes—fatty acid biosynthesis and transcription factor genes in ABT larvae

**Fig. 1:**
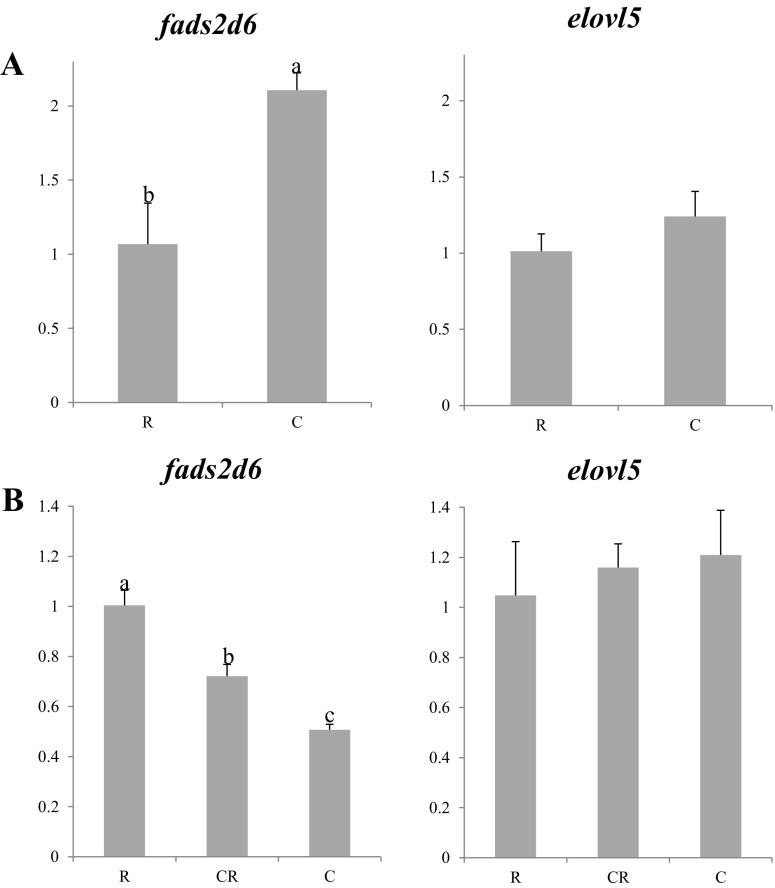
Nutritional regulation of *fads2d6* and *elovl5* gene transcription in whole larvae of Atlantic bluefin tuna fed different dietary treatments in the 2013 (**a**) or 2014 (**b**) trials. Feeds were either enriched rotifers (*R*), nauplii of copepods (*C*), or co-feeding of both live preys (*CR*). Values are normalized expression ratios, corresponding to an average of six individuals (*n* = 6) with standard errors (SEM). Different *superscript letters* denote differences between the dietary treatments. *fads2d6* delta-6 fatty acyl desaturase; *elovl5* fatty acyl elongase 5

Differing results were observed regarding expression of *fads2d6* between the two experiments. In this regard, in the 2013 experiment, the larvae fed copepods showed higher expression of *fads2d6* compared to the larvae fed rotifers (Fig. 1a). In contrast, expression of *fads2d6* was the lowest in copepod-fed larvae in 2014 (Fig. 1b). No differences were observed regarding *elovl5* expression, although there was a tendency for higher expression in copepod-fed larvae (Figs. 1a, b).

**Fig. 2:**
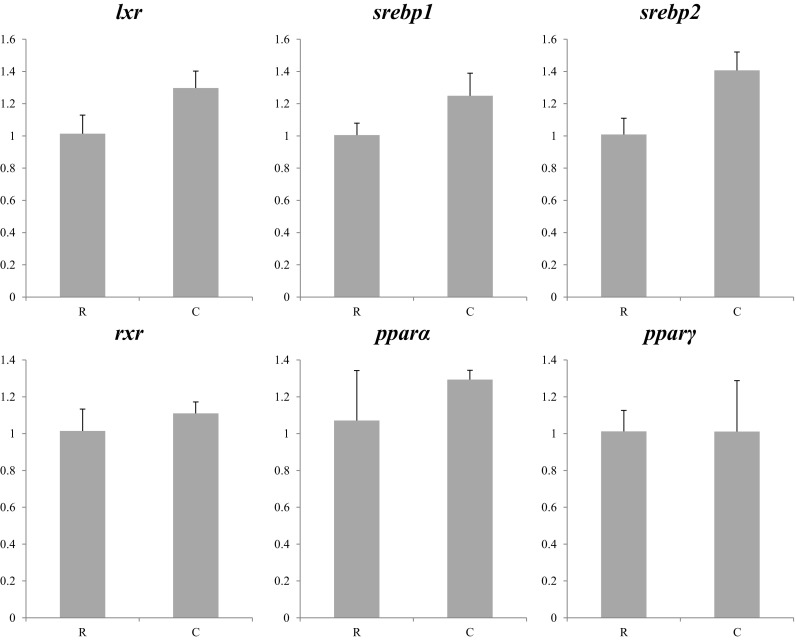
Nutritional regulation of transcription factor genes in whole larvae of Atlantic bluefin tuna fed different dietary treatments in the 2013 trial. Feeds were either enriched rotifers (*R*) or nauplii of copepods (*C*). Values are normalized expression ratios, corresponding to an average of six individuals (*n* = 6) with standard errors (SEM). Different *superscript letters* denote differences between the dietary treatments. *lxr* liver X receptor; *srebp1* sterol regulatory element-binding protein 1; *srebp2* sterol regulatory element-binding protein 2; *rxr* retinoid X receptor; *pparα* peroxisome proliferator-activated receptor alpha; *pparγ* peroxisome proliferator-activated receptor gamma

**Fig. 3:**
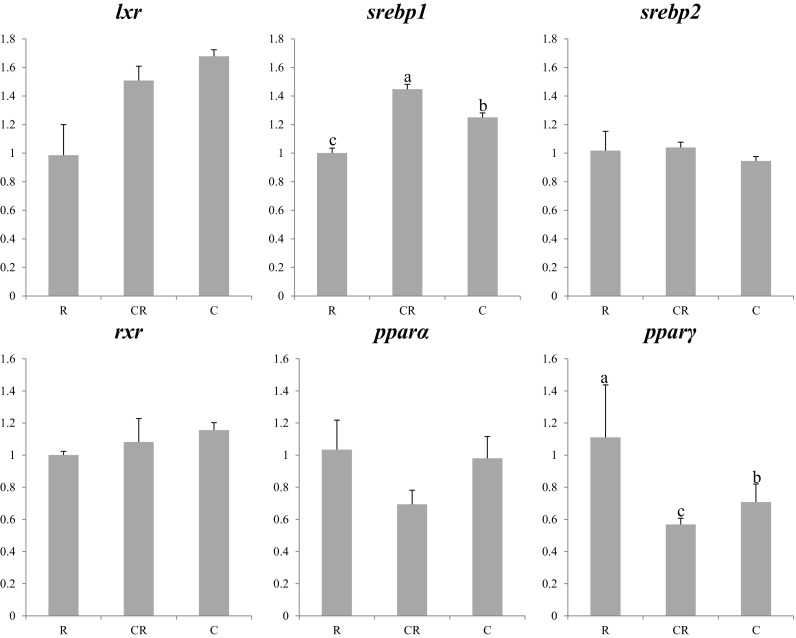
Nutritional regulation of transcription factor genes in whole larvae of Atlantic bluefin tuna fed different dietary treatments in the 2014 trial. Feeds were either enriched rotifers (*R*), nauplii of copepods (*C*), or co-feeding of both live preys (*CR*). Values are normalized expression ratios, corresponding to an average of six individuals (*n* = 6) with standard errors (SEM). Different *superscript letters* denote differences between the dietary treatments. *lxr* liver X receptor; *srebp1* sterol regulatory element-binding protein 1; *srebp2* sterol regulatory element-binding protein 2; *rxr* retinoid X receptor; *pparα* peroxisome proliferator-activated receptor alpha; *pparγ* peroxisome proliferator-activated receptor gamma

In the 2013 experiment, no differences in expression were observed in transcription factors between larvae fed copepods or rotifers (Fig. 2), whereas some genes presented differing expression among larvae fed the three dietary treatments in 2014 (Fig. 3). In this regard, *srebp1* expression was the highest in co-fed larvae, with the larvae fed rotifers displaying the lowest expression of this gene (Fig. 3). Differences between larvae fed the three dietary treatments in 2014 were also observed for *pparγ* with lowest expression displayed by co-fed larvae and highest expression observed in larvae fed rotifers (Fig. 3). Although no significant differences were found in *lxr* or *rxr* mRNA levels, the same expression patterns were observed in larvae from the two trials, with low expression of these TFs in larvae fed rotifers compared to larvae fed copepod, with co-fed larvae showing intermediate expression levels in the 2014 trial (Figs. 2 and 3).

**Fig. 4:**
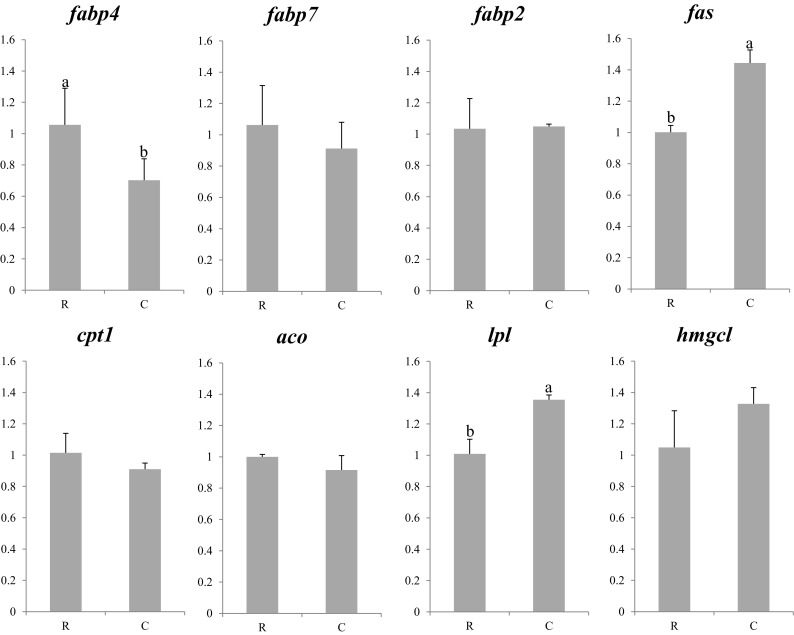
Nutritional regulation of lipid metabolism gene transcription in whole larvae of Atlantic bluefin tuna fed different dietary treatments in the 2013 trial. Feeds were either enriched rotifers (*R*) or nauplii of copepods (*C*). Values are normalized expression ratios, corresponding to an average of six individuals (*n* = 6) with standard errors (SEM). Different *superscript letters* denote differences between the dietary treatments. *fabp*4 fatty acid binding protein 4 (adipocyte); *fabp7* fatty acid binding protein 7 (brain-type); *fabp2* fatty acid binding protein 2 (intestinal); *fas* fatty acid synthase; *cpt1* carnitine palmitoyl transferase I; *aco* acyl coA oxidase; *lpl* lipoprotein lipase; *hmgcl* 3-hydroxy-3-methylglutaryl-CoA lyase

**Fig. 5:**
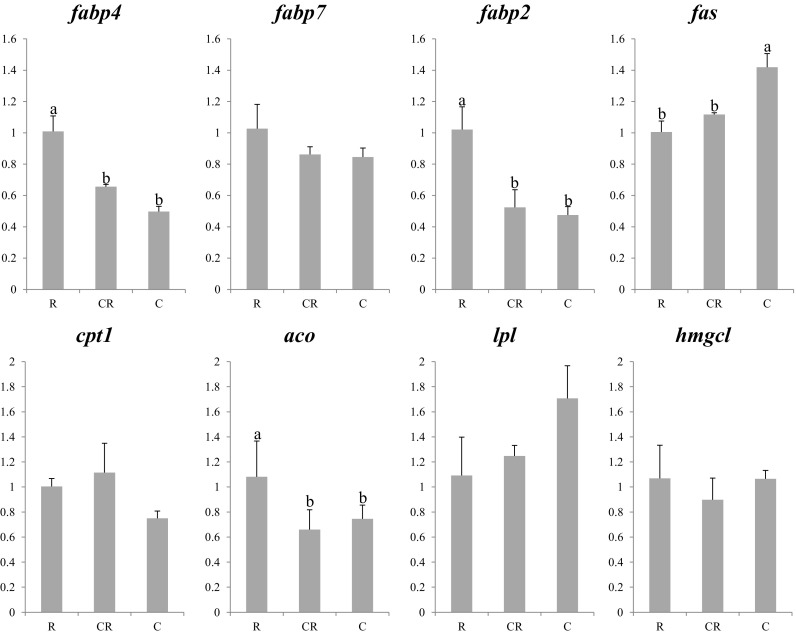
Nutritional regulation of lipid metabolism gene transcription in whole larvae of Atlantic bluefin tuna fed different dietary treatments in the 2014 trial. Feeds were either enriched rotifers (*R*), nauplii of copepods (*C*), or co-feeding of both live preys (*CR*). Values are normalized expression ratios, corresponding to an average of six individuals (*n* = 6) with standard errors (SEM). Different *superscript letters* denote differences between the dietary treatments. *fabp4* fatty acid binding protein 4 (adipocyte); *fabp7* fatty acid binding protein 7 (brain-type); *fabp2* fatty acid binding protein 2 (intestinal); *fas* fatty acid synthase; *cpt1* carnitine palmitoyl transferase I; *aco* acyl coA oxidase; *lpl* lipoprotein lipase; *hmgcl* 3-hydroxy-3-methylglutaryl-CoA lyase

The expression profiles of lipid homeostasis genes are shown in Figs. 4 and 5. In general, expression of *fabp*s showed relatively stable patterns in both years, with higher expression in larvae fed rotifers than in larvae fed copepods, or co-fed larvae. However, significant differences were only observed in *fabp4* expression for 2013 larvae and in *fabp4* and *2* for 2014 larvae. The expression of *fas* also showed differences between treatments in both years, with highest expression in larvae fed copepods, and there was no difference between larvae fed solely on rotifer or co-fed rotifers and copepods in 2014. In contrast to the 2013 experiment, *aco* displayed differences in expression among dietary treatments in larvae in the 2014 trial. In this case, larvae fed rotifers showed the highest expression with no differences between copepod-fed or co-fed larvae. Larvae fed rotifers showed the lowest expression of *lpl* in 2013 with a similar but non-signifant tendency observed in 2014. No differences among treatments in either year were found in the expression of *fabp7*, *cpt1*, or *hmgcl*.

### Tissue distribution of lipid and fatty acid metabolism and transcription factor genes in adult ABT

**Fig. 6:**
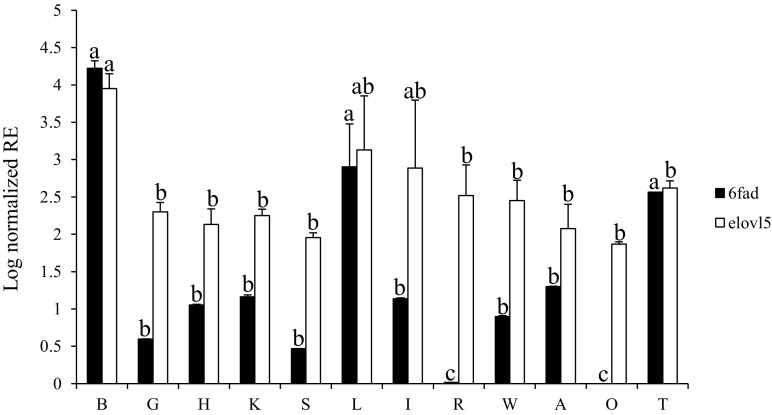
Tissue distribution of *fads2d6* (*black*) and *elovl5* (*white*) transcripts. The transcript expression level was determined by qPCR in 12 tissues. Values correspond to the log-normalized (*ef1α*) relative expression (RE) of the target genes in each tissue. For comparison, the expression level of *fads2d6* in ovary, which was the lowest, was defined as 1. The results represent the average of six individuals (*n* = 6) with standard errors (SEM). *B* brain; *G* gills; *H* heart; *K* kidney; *S* spleen; *L* liver; *I* intestine; *R* red muscle; *W* white muscle; *A* adipose tissue; *O* ovary; *T* testis

The tissue expression profiles showed that both LC-PUFA biosynthetic pathway genes (*elovl5* and *fads2d6*) were expressed in all tissues examined, with the highest expression levels in brain followed by liver and testis (Fig. 6). The *elovl5* transcript was more abundant than *fads2d6* in all other tissues, with particularly low expression of *fads2d6* in red muscle and ovaries (Fig. 6).

**Fig. 7:**
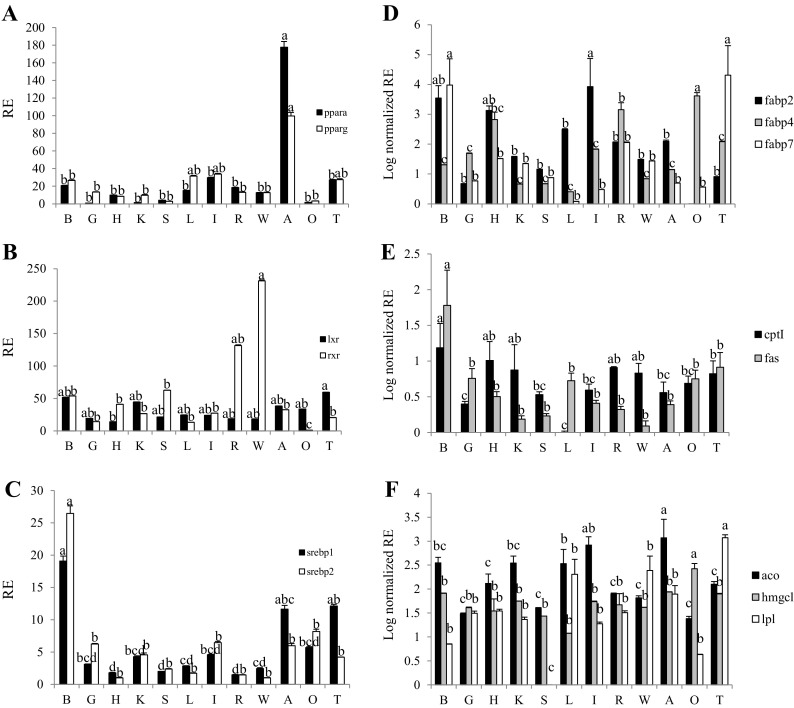
Tissue distribution of pparα and *γ* (**a**), lxr and rxr (**b**), srebp1 and 2 (**c**), fabp genes (**d**), cpt1 and fas (**e**) and aco, and hmgcl and lpl (**f**). The transcript expression level was determined by qPCR in 12 tissues. Values correspond to the normalized (*ef1α*) relative expression (RE) of the target genes in each tissue. For comparison, the expression level of *pparα* in gills (**a**), *rxr* in ovary (**b**), *srebp2* in white muscle (**c**), *fabp2* in ovary (**d**), *cptI* in liver (**e**), and *lpl* in spleen (**f**) were lowest and defined as 1. The results represent the average of six individuals (*n* = 6) with standard errors (SEM). *B* brain; *G* gills; *H* heart; *K* kidney; *S* spleen; *L* liver; *I* intestine; *R* red muscle; *W* white muscle; *A* adipose tissue; *O* ovary; *T* testis. *Different letters* indicate significant differences among tissues for each of the genes (*P* < 0.05)

Regarding transcription factors, *pparα* and *pparγ* showed parallel expression, with adipose tissue exhibiting the highest relative copy number (Fig. 7a) of all the evaluated tissues, followed by intestine > testis > liver. The expression of *lxr* was surprisingly low in liver, with highest expression in testis, brain, and kidney. Similarly, *rxr* was poorly expressed in liver with higher levels of expression in both white and red muscle, followed by spleen and brain (Fig. 7b). The rank order of expression of *srebp1* was brain > testis > ovary > intestines > kidney > gill > liver > white muscle > spleen > heart and red muscle (Fig. 7c). For *srebp2*, the highest expression was also observed in brain followed by testis and adipose tissue with lowest expression in heart and white muscle (Fig. 7c).

The expression of *fabp2* (intestinal isoform) was highest in intestine, followed by brain and heart, with lower levels of expression in liver, red muscle, adipose tissue, and kidney (Fig. 7d). In contrast, the adipocyte isoform *fabp4* showed highest expression in ovaries with liver showing the lowest values, with red muscle, heart, and adipose tissue showing intermediate values (Fig. 7d). The brain isoform *fabp7* showed highest expression levels in brain and testis, followed by red and white muscle, heart, and kidney, with lowest values in liver (Fig. 7d). The highest expression of *cptI* was in brain and the lowest expression in liver (Fig. 7e). Similarly, the relative copy number for *fas* was highest in brain followed by gonads, gill, and liver, with white muscle showing the lowest expression (Fig. 7e). The expression of *aco* was highest in adipose tissue and intestine, followed by liver, kidney, and brain (Fig. 7f). The expression of *hmgcl* was highest in ovary followed by adipose tissue, brain, and testis, with lowest expression in liver (Fig. 7f). Finally, the expression of *lpl* was highest in testis and lowest in ovary, with liver and white muscle, adipose tissue, heart, gills and red muscle, kidney, and intestine showing intermediate levels of expression (Fig. 7f).

## Discussion

The present study reports on the expression of fatty acid biosynthesis, lipid metabolism, and transcription factor genes in ABT larvae during first feeding with different live prey, specifically enriched rotifers versus copepod nauplii and copepodites. Due to the short spawning season of ABT in natural conditions (only June and July) and the scarce availability of fertilized viable eggs, trials on first feeding ABT larvae were performed over two periods corresponding to 2013 and 2014 spawning seasons.

Bluefin tuna larvae predate copepods in the wild (Uotani et al. 1990), and, in general, marine fish larvae fed on copepods grow faster than larvae fed enriched rotifers or *Artemia*, as has been shown with cod (*Gadus morhua*; Hamre 2006), grouper (*E. coioides*; Toledo et al. 1999), or turbot (*Scophtalmus maximus*; Witt et al. 1984). In addition, in a previous study, ABT larvae fed on *A. tonsa* copepods showed high growth and survival rates (Yufera et al. 2014). Our hypothesis was that copepods may be a better live prey than *Artemia* due to their high DHA and DHA/EPA ratio. Furthermore, although n-3 LC-PUFA levels can be similar in enriched *Artemia* and copepods, they are found within the neutral lipids in the former (Sargent et al. 1999), whereas they are predominantly found in polar lipids (e.g., phospholipids) in copepods and may be more easily assimilated by the larvae (Shields et al. 1999).

In the present study, ABT larval survival was similar in both years, with copepod-fed larvae (and co-fed larvae) showing higher survival than larvae fed rotifers, probably indicating that copepods were a more suitable live prey for larvae of this fast-growing fish species. Conversely, growth differed in the 2 years, with larvae fed rotifers showing higher growth rates than those fed copepods in 2013, but the opposite in 2014. Rotifers supplied more lipid per dry mass than copepods in both years, so this on its own does not correlate with survival or growth. Similarly, differences in lipid class composition between rotifers and copepods were essentially the same in both years but, in contrast, fatty acid compositions of the live feeds varied. Thus, in both 2013 and 2014 trials, copepods supplied considerably more DHA on a relative basis than rotifers. The DHA/EPA ratio was similar between copepod- and rotifer-fed larvae in 2013, being higher in copepods than in rotifers in 2014. This may suggest that the absolute DHA level may be important for the survival of ABT larvae but that the DHA/EPA ratio may be relatively more important for larval growth. However, neither DHA level nor the DHA/EPA ratio on their own can explain the variation between the 2 years. It has been shown that broodstock nutrition, particularly dietary lipid, can greatly affect larval survival and growth (Sargent 1995; Wiegand 1996; Izquierdo et al. 2001). Although ABT broodstock were not evaluated in the present trials, it is likely that fertilized eggs belonging to different spawning groups lead to differing results in larval performance between the 2 years. Additionally, broodstock dietary differences between the 2 years could also have influenced larval performance. The present trial aimed to provide further insight into the effects of nutrition on ABT larval performance by investigating lipid metabolism by determining expression patterns of some key genes, rather than focusing on associations between dietary compositions and resultant larval compositions. To the authors’ knowledge, this is the first report to evaluate expression of lipid metabolism genes in ABT larvae under different feeding regimes at 14 dah.

One obvious and potentially key pathway to investigate is LC-PUFA biosynthesis. Modulation of *fads2d6* was observed in both years, but with differing direction of expression, which may be related to differences in fatty acid profiles of the live prey as differences in the levels of n-3 LC-PUFA were observed between the 2 years. In contrast, expression of *elovl5* in the present study showed no significant regulation. Generally, up-regulation of *fads2d6* expression has been observed in fish fed low dietary levels of n-3 LC-PUFA, for instance when high levels of vegetable oil are included in feeds (Morais et al. 2012; Betancor et al. 2015). This is what was observed in ABT larvae fed enriched rotifers and copepods in 2013, but not in 2014. However, the activities of *fads2d6* and *elovl5* in marine fish have been the subject of discussion and speculation as most marine species have only low capacity for LC-PUFA synthesis from C_18_ PUFA precursors such as 18:3n-3, probably as a consequence of them obtaining high levels of n-3 LC-PUFA in their natural diet (Tocher 2010). However, an alternative role for the *fads2d6* and *elovl5* enzymes in marine fish has been suggested based on a requirement to maintain membrane DHA levels, especially in neural tissues, at times of high demand such as embryonic and larval development (Tocher et al. 2006; Zheng et al. 2009; Mohd-Yusof et al. 2010). Consistent with this, the present study has shown that both *fads2d6* and *elovl5* were expressed at highest levels in adult tuna brain, showing the typical marine species expression pattern such as previously shown in Atlantic cod (*G. morhua*) and cobia (*Rachycentron canadum*) (Tocher et al. 2006; Zheng et al. 2009; Xue et al. 2014), given that freshwater species tend to have higher levels of expression in liver than in brain (Oboh et al. 2016). The alternative role of these genes in marine fish in maintaining (neural) tissue DHA levels (by desaturation and elongation of EPA) may at least partly explain the differences in expression observed. Thus, in 2013, the high level of dietary EPA in copepods resulted in higher expression of *fads2d6* in ABT larvae. In 2014, the level of EPA was lower, and DHA higher, in copepods than in rotifers (and copepods in 2013), and this high DHA/EPA ratio in copepods appeared to inhibit expression of *fads2d6* in ABT larvae fed them. Therefore, the higher DHA levels found in copepod-fed ABT larvae in 2013 than in copepods themselves may be a consequence of both desaturation of EPA and/or selective retention of this fatty acid.

A previous study evaluating the expression of both *fads2d6* and *elovl5* in unfed ABT larvae showed up-regulation of these genes during early development (Morais et al. 2011). Consistent with the increased expression, the DHA/EPA ratio in the ABT larvae also increased during development, but retention of DHA in polar lipids and selective catabolism of EPA also offer alternative mechanisms. Irrespective of the primary mechanism for the increased DHA/EPA ratio in the study with unfed larvae, the increasing expression of *fads2d6* and *elovl5* suggested that endogenous biosynthesis of DHA may be also important during normal early development in ABT larvae at early feeding stages (Morais et al. 2011). In natural development with exogenous feeding, the increased expression of *fads2d6* and *elovl5* genes and the consequent increased conversion of EPA to DHA would be an important pathway to help satisfy the high requirement for DHA necessary for the rapid development of neural tissue (brain and eye) during early exogenous feeding (Mourente 2003). The present study has shown how the dietary DHA/EPA ratio may also modulate the expression of *fads2d6* in ABT larvae and confirmed that the LC-PUFA biosynthesis pathway may be especially important in neural tissues in tuna.

PPARs are ligand-activated members of the nuclear hormone receptor family of TFs (Feige et al. 2006) that are encoded by three genes in mammals, and homologs of all three have been characterized in fish (Leaver et al. 1998; Boukouvala et al. 2004; Diez et al. 2007). Different isoforms of PPAR regulate lipid deposition and mobilization, and lipid metabolism including LC-PUFA synthesis, and activating ligands include FA and their metabolites (Kennedy et al. 2006; Leaver et al. 2006; Agawa et al. 2012). LXRs are encoded by two distinct genes in mammals (Zhao and Dahlman-Wright 2010), but only one in fish and the salmon homolog has been characterized (Cruz-Garcia et al. 2009). LXRs act to regulate the formation of bile acids from cholesterol and are activated by oxysterol ligands, but they are also involved in the regulation of SREBPs that are encoded by two genes in mammals and both have homologs in fish (Minghetti et al. 2011). SREBP-1 preferentially regulates FA and LC-PUFA synthesis whereas SREBP-2 regulates the expression of genes involved in cholesterol synthesis including HMGCoA reductase. Target genes for SREBP-1 include FA synthase (*fas*) and glycerol phosphate acyltransferase involved in the synthesis of TAG and phospholipids. In addition, SREBP-1 has been implicated in the synthesis of LC-PUFA through regulation of *fad* and *elovl* genes (Horton et al. 2002; Matsuzaka et al. 2002; Botolin et al. 2006). Regulatory loops involving these TFs are particularly important in integrating metabolism, and so SREBP-1 is a target gene of LXRα, and LXRα is a target gene of PPARα (Repa et al. 2000; Chawla et al. 2001; Qin et al. 2009). In addition, PUFA act to activate PPARα and to suppress SREBPs (Worgall et al. 1998; Hihi et al. 2002). Recently, it was shown that similar regulatory interactions exist in fish cells (Minghetti et al. 2011; Carmona-Antoñanzas et al. 2014). Therefore, expression of TFs involved in lipid metabolism were also evaluated in the present study.

Although no significant differences were observed in the 2013 trial, *srebp1* and *pparγ* showed differential expression with diet in the 2014 trial with *srebp1* expression down-regulated in larvae fed rotifers, which also displayed an up-regulation of *fads2d6*. In mammals, *srebp1* increases the expression of genes involved in fatty acid synthesis, including *fas*, *fads2d6*, and *elovl5* (Horton et al. 2007). The present data in ABT larvae contradicts previously observed in mammals (Matsuzaka et al. 2002) and in fish (Betancor et al. 2014), where concomitant up-regulation of both genes had been described. Therefore, in contrast to what may have been expected, the lower levels of PUFA found in rotifer-fed larvae compared to copepod-fed larvae did not increase *srebp1* expression, but actually reduced the expression. On the other hand, *pparγ*, which controls lipid accumulation and regulates adipogenesis and osteogenesis (Nedergaard et al. 2005; Ji et al. 2011; Agawa et al. 2012), was up-regulated in the 2014 trial in rotifer-fed larvae, which also showed reduced TAG, SE, and MUFA contents as well as increased phospholipid levels. Similar results were observed in unfed turbot larvae where an up-regulation in *pparγ* was found when the yolk sac was completely absorbed accompanied by an up-regulation of *fads2d6* (Cunha et al. 2013), as was also observed in the present study. This may indicate that fatty acid desaturases can be target genes for *pparγ* as has been previously suggested for teleosts (Cunha et al. 2013). Additionally, PPAR*γ* plays an important role in regulating lipid metabolism in mature adipocytes (Lehrke and Lazar 2005) and the highest expression levels of *pparγ* were found in adipose tissue in the present study, consistent with what has been previously reported in other fish species (Tsai et al. 2008; Zheng et al. 2015). The tissue expression of *pparα* was also similar to that of the Japanese sea bass (*L. japonicus*), with the highest expression levels in adipose tissue, although this does not follow the pattern of other teleost species (Tsai et al. 2008; Zhao et al. 2011).

In mammals, target genes directly regulated by *pparγ* include genes that favor uptake of circulating fatty acids by adipocytes (Schoonjans et al. 1996; Frohnert et al. 1999; Chui et al. 2005) and others that promote recycling rather than export of intracellular fatty acids (Guan et al. 2002; Hibuse et al. 2005). These paradoxical effects on adipocyte biology means that, apart from enhancing fatty acid deposition as PPARα does, PPARγ can lead to increased fatty acid oxidation (Lehrke and Lazar 2005). This may explain why higher *pparγ* expression in larvae fed rotifers in 2014 trial was associated with up-regulation of *aco*, an oxidoreductase that participates in β-oxidation, as well as down-regulation of *fas*, involved in fatty acid synthesis. Although rotifer-fed larvae displayed slightly higher lipid content (albeit not significant), they also had a smaller size compared to larvae fed copepods or a combination of copepods and rotifers, which could mean higher energy requirements for growth that could, in turn, explain up-regulation in *pparγ*. Similarly, *ppar*γ was correlated to de novo fatty acid synthesis (*fas*) and also to phospholipid hydrolysis (hepatic lipase) in unfed turbot larvae (Cunha et al. 2013).

Consistent with the above, *fabp4* and *fabp2*, carrier proteins involved in fatty acid uptake, transport, and metabolism (Glatz and van der Vusse 1996), were also up-regulated in rotifer-fed larvae (2014), perhaps reflecting increased uptake and accumulation of lipid into larval tissues. Similarly, Senegalese sole (*Solea senegalensis*) larvae showed differential regulation of *fabp2* expression when fed different levels of n-3 LC-PUFA, particularly EPA (Darias et al. 2012), which in turn translated into higher liver lipid deposition (Boglino et al. 2012). It is notable therefore that EPA was also higher in enriched rotifers than copepods in the 2014 trial in the present study. However, a recent study in Senegalese sole larvae showed no regulation of *fabp2* expression when larvae were fed enriched *Artemia*, whereas up-regulation of *fabp1* and *fabp3* was observed in larvae fed high levels of n-3 LC-PUFA (Bonacic et al. 2016), which may indicate differential regulation of *fabp* at different developmental stages (André et al. 2000). In the 2013 trial, rotifer-fed larvae, which showed highest growth but lowest survival, also showed up-regulation of *fabp4* and down-regulation of *fas* expression, although no changes were observed in the expression of any TF. Additionally, these larvae showed down-regulation of *lpl*, a lipase highly expressed in muscle and liver of ABT as shown in the present study and that hydrolyzes TAG in plasma lipoproteins and supplies free fatty acids for deposition in adipose tissue or for oxidation in other tissues (Nilsson-Ehle et al. 1980; Kersten 2014). High levels of *lpl* expression and activity have been associated with increased lipid utilization in darkbarbel catfish (*Pelteobagrus vachelli*) larvae fed high-lipid diets (Zheng et al. 2010). Thus, ABT larvae fed rotifers in 2013 that showed enhanced *fabp4* and reduced *fas* expression may be compensating for reduced growth, and reduced *lpl* expression may reflect lower lipid utilization.

The tissue distribution profiles of genes is essential to improve our understanding of the roles of these lipid metabolic pathways in tuna physiology. In the present study, seven different tissues from both male and female adult ABT were screened. Brain displayed the highest expression levels of *srebp1*, *srebp2*, and *fas*, which was consistent with studies in other marine teleost species (Dong et al. 2015; Zhang et al. 2016). The expression of *cpt1* was also highest in brain, followed by muscular tissues with the lowest expression in liver, which was similar to the tissue expression profile observed previously for *cpt1β* in darkbarbel catfish (Zheng et al. 2013). Similarly, gilthead sea bream (*Sparus aurata*) showed higher expression levels of *cpt1* in heart and red and white muscle, although brain showed low levels of expression (Boukouvala et al. 2010). Fatty acid-binding proteins followed the expected pattern with *fabp2* showing higher expression level in intestine and *fabp7* in brain (Yamamoto et al. 2009). Surprisingly, *fabp4*, which is considered the adipocyte fabp, showed higher levels of expression in ovary and red muscle than adipose tissue in contrast to the tissue expression pattern described in mammals (Yamamoto et al. 2009). Previous studies in freshwater fish have reported highest expression levels of *aco* in liver, intestine, kidney, and brain (Morais et al. 2007; He et al. 2014), whereas adipose tissue and intestine showed higher levels of *aco* expression in ABT tissues, suggesting species or environmental differences. Similarly, the tissue expression pattern of *lpl* appears to differ among teleosts with highest expression levels in testis, liver, and white muscle in the present study compared with liver (Zheng et al. 2013; Feng et al. 2014) and adipose tissue (Cheng et al. 2009) in other species. The highest expression levels of *hmgcl* in ovary agree with the results obtained in the crab *Scylla paramamosain* (Zhao et al. 2015).

In conclusion, the present study with ABT larvae showed that copepods are a better live prey for ABT for first feeding (particularly in 2014 trial), based on enhanced growth and survival. Differences in the expression patterns of lipid metabolism genes were observed between the two trials. Some of the responses in lipid gene expression could be a consequence of dietary (prey) lipid and FA composition, but there was no obvious direct correlation between gene expression and growth or survival. Differences in performance and metabolism among larval groups between the trials could also partly reflect differences in broodstock and their nutrition in the 2 years. Up-regulation of *fabp4*, *pparγ*, and *aco* in rotifer-fed larvae may be associated with a compensatory response to reduced growth, whereas lower expression of *lpl* could denote reduced lipid utilization. Although effects of nutrient profiles of enriched rotifers can be found in the literature of bluefin tuna larval production, no study has reported lipid requirements, including EPA and DHA requirements, of larval tuna during early feeding stages (rotifer and/or copepod feeding period) (Buentello et al. 2016). Therefore, further studies are required to investigate lipid requirements, lipid accumulation, and lipid metabolism during development of ABT larvae. The expression patterns of lipid metabolism genes in adult ABT tissues provided insight to the physiology of lipid metabolism in tuna and showed species-related differences, particularly compared to freshwater fish. Special importance should be given to the expression analysis of genes related to lipid metabolism and its regulation, combined with biochemical studies of tuna lipid metabolism in order to develop optimal feeds to facilitate the commercial culture of this iconic species.

## Electronic supplementary material

ESM 1 (DOCX 19 kb)

## Abbreviations

ABT: Atlantic bluefin tuna
*aco*: Acyl coA oxidase
ARA: Arachidonic acid (20:4n-6)
C: Free cholesterol
*cpt1*: Carnitine palmitoyl transferase I
dah: Days after hatch
DHA: Docosahexaenoic acid (22:6n-3)
EFA: Essential fatty acid
EPA: Eicosapentaenoic acid (20:5n-3)
*elovl5*: Fatty acyl elongase 5
*fabp2*: Fatty acid binding protein 2 (intestinal) fatty acid
*fabp*4: Fatty acid binding protein 4 (adipocyte)
*fabp7*: Fatty acid binding protein 7 (brain-type)
*fads2d6*: Delta-6 fatty acyl desaturase
FAME: Fatty acid methyl ester
*fas*: Fatty acid synthase
*hmgcl*: 3-Hydroxy-3-methylglutaryl-CoA lyase
HPTLC: High performance thin-layer chromatography
LC-PUFA: Long-chain polyunsaturated fatty acid
*lpl*: Lipoprotein lipase
*lxr*: Liver X receptor
MUFA: Monounsaturated fatty acid
PBT: Pacific bluefin tuna
PC: Phosphatidylcholine
PE: Phosphatidylethanolamine
PI: Phosphatidylinositol
*pparα*: Peroxisome proliferator-activated receptor alpha
*pparγ*: Peroxisome proliferator-activated receptor gamma
PS: Phosphatidylserine
qPCR: Quantitative real-time PCR
*rxr*: Retinoid X receptor
SE: Steryl ester
*srebp1*: Sterol regulatory element-binding protein 1
*srebp2*: Sterol regulatory element-binding protein 2
TAG: Triacylglycerol
TF: Transcription factor
