# Supplementary material for: Lipid metabolism-related gene expression pattern of Atlantic bluefin tuna (Thunnus thynnus L.) larvae fed on live prey
Source: Fish Physiol Biochem. 2016 Nov 4;43(2):493–516. doi: 10.1007/s10695-016-0305-4 (PMC5374188; doi:10.1007/s10695-016-0305-4)
Supplement: Supplementary file 1 — (DOCX 19 kb) [file 10695_2016_305_MOESM1_ESM.docx]

| Aim | Name | Sequence (5’-3’) | Amplicon size (bp) | Tm°C | Accesion no |
| --- | --- | --- | --- | --- | --- |
| *ORF* | *srebp1* | F: GGCTGGATGGATTGGATGCT | 758 | 57 | - |
|  |  | R: TGGCTGTAGATGCTTTCTTT |  |  |  |
|  | *srebp2* | F: CTGGGCCACCATCTCTCCTG | 954 | 62 | - |
|  |  | R: GTGGGACTGGCTCCAGCCAT |  |  |  |
|  | *lxr* | F: TGTACATGCGCCGCAAGTG | 589 | 62 | - |
|  |  | R: AACTCAAAGATGGGGTTGA |  |  |  |
|  | *pparα* | F: GTGGGCATGTCCCACAACGC | 683 | 60 | - |
|  |  | R: CAGCAGATGATAGCAGCAC |  |  |  |
|  | *lpl* | F: GCCAACGTCATCGTGGTGGA | 387 | 60 | - |
|  |  | R: GGGGTAAATGTCTATGTGGC |  |  |  |
|  | *cpt1* | F: TATGTCAGTGACTGGTGGGA | 251 | 60 | - |
|  |  | R: GTGTTGAACATACGCTCGTA |  |  |  |
| *qPCR* | *elovl5* | F: CCACGCTAGCATGCTGAATA | 236 | 60 | HQ214237 |
|  |  | R: ATGGCCATATGACTGCACAC |  |  |  |
|  | *fads2d6* | F: CCGTGCACTGTGTGAGAAAC | 152 | 60 | HQ214238 |
|  |  | R: CAGTGTAAGCGATAAAATCAGCTG |  |  |  |
|  | *pparα* | F: TGGTCATGGAGGTGGAAGAC | 152 | 60 | KU900605 |
|  |  | R: ATGGATGACGAAAGGAGGGG |  |  |  |
|  | *pparγ* | F: ACCTGACCAACATGGACTAC | 118 | 60 | AB574331 |
|  |  | R: GAGAAAACAGGACTGTCAGC |  |  |  |
|  | *lxr* | F: CACACTGGATCCACAACAGC | 192 | 58 | KU900602 |
|  |  | R: ATCTCCTGCACCGACATGAT |  |  |  |
|  | *rxr* | F: TGAGGGAAAAGGTCTACGCA | 212 | 59 | EC092909 |
|  |  | R: TGTGATCTGATGTGGTGCCT |  |  |  |
|  | *srebp1* | F: CCAGCTACACATGACAGGGA | 153 | 59 | KU900604 |
|  |  | R: GCTTTGACCCTTAGAGCTGC |  |  |  |
|  | *srebp2* | F: AGATCCAGTGAGTCGTTGGT | 212 | 60 | KU900603 |
|  |  | R: CTACAGCCCCTTCTCCCTTC |  |  |  |
|  | *fabp2* | F: CGCAGCGAGAATTATGACAA | 244 | 55 | EG999641 |
|  |  | R: AGCATGTCACCCTCCATCTC |  |  |  |
|  | *fabp4* | F: ACTGCAATGACCGAAAGACC | 175 | 55 | EC092703 |
|  |  | R: CCTCCTTTCCGTAGGTCCTC |  |  |  |
|  | *fabp7* | F: CCTACACCTGATGACCGACA | 212 | 55 | EG999669 |
|  |  | R: GCTGGGATGATTTGCTCATT |  |  |  |
|  | *cptI* | F: TGGAGGCTGTCCACCAGTCA | 211 | 60 | KU900606 |
|  |  | R: TGCTGGAGATGTGGAAGTTG |  |  |  |
|  | *lpl* | F: CCGAAAGAACCGCTGCAATA | 212 | 59 | - |
|  |  | R: GATCCTCCTTCTCTCCGTGG |  |  |  |
|  | *fas* | F: ATACCGTGGCAATGTAACGC | 188 | 59 | - |
|  |  | R: GTGAGCTGTGGATGATGCTG |  |  |  |
|  | *aco* | F: AGCGCTATGACCAGGCTATT | 164 | 59 | - |
|  |  | R: GTACAGGGTTGGGAGGAACA |  |  |  |
|  | *hmgl* | F: CGTGCCAACAGAGACGAAAA | 173 | 59 | EC092121 |
|  |  | R: GGGTGAGGACTGGGTAAGAC |  |  |  |
|  | *ef1a* | F: CCCCTGGACACAGAGACTTC | 119 | 60 | FM995222 |
|  |  | R: GCCGTTCTTGGAGATACCAG |  |  |  |
|  | *bactin* | F: ACCCACACAGTGCCCATCTA | 155 | 61 | GU046791 |
|  |  | R: TCACGCACGATTTCCCTCT |  |  |  |

**Supplementary Table 1**.- Sequence, annealing temperature (Tm) and size of the fragment produced by the primer pairs used for quantitative PCR (qPCR).

*elovl5*, fatty acyl elongase 5; *fads2d6*, delta-6 fatty acyl desaturase; *pparα*, peroxisome proliferator-activated receptor alpha; *pparγ*, peroxisome proliferator-activated receptor gamma; *lxr*, liver X receptor; *rxr*, retinoid X receptor; *srebp1*, sterol regulatory element-binding protein 1; *srebp2*; sterol regulatory element-binding protein 2; *fabp2*, fatty acid binding protein 2 (intestinal); *fabp4*, fatty acid binding protein 4 (adipocyte); *fabp7*, fatty acid binding protein 7 (brain-type); *cptI*, carnitine palmitoyl transferase I; *lpl*, lipoprotein lipase; *fas*; fatty acid synthase; *aco*, acyl coA oxidase; *hmgl*; 3-hydroxy-3-methylglutaryl-CoA lyase; *ef1α*, elongation factor 1 alpha.
